# Timely Pulmonary Tuberculosis Diagnosis Based on the Epidemiological Disease Spectrum: Population-Based Prospective Cohort Study in the Republic of Korea

**DOI:** 10.2196/47422

**Published:** 2024-04-01

**Authors:** Yousang Ko, Jae Seuk Park, Jinsoo Min, Hyung Woo Kim, Hyeon-Kyoung Koo, Jee Youn Oh, Yun-Jeong Jeong, Eunhye Lee, Bumhee Yang, Ju Sang Kim, Sung-Soon Lee, Yunhyung Kwon, Jiyeon Yang, Ji yeon Han, You Jin Jang, Jinseob Kim

**Affiliations:** 1 Division of Pulmonary, Allergy and Critical Care Medicine Department of Internal Medicine Hallym University Kangdong Sacred Heart Hospital Seoul Republic of Korea; 2 Division of Pulmonary Medicine Department of Internal Medicine Dankook University College of Medicine Cheonan Republic of Korea; 3 Division of Pulmonary and Critical Care Medicine Department of Internal Medicine Seoul St. Mary's Hospital, College of Medicine, The Catholic University of Korea Seoul Republic of Korea; 4 Division of Pulmonary and Critical Care Medicine Department of Internal Medicine Incheon St. Mary’s Hospital, College of Medicine, The Catholic University of Korea Incheon Republic of Korea; 5 Division of Pulmonary and Critical Care Medicine Department of Internal Medicine Ilsan Paik Hospital, Inje University College of Medicine Ilsan Republic of Korea; 6 Division of Pulmonary, Allergy, and Critical Care Medicine Department of Internal Medicine Korea University College of Medicine, Korea University Guro Hospital Seoul Republic of Korea; 7 Division of Pulmonary and Critical Care Medicine Department of Internal Medicine Dongguk University Ilsan Hospital Ilsan Republic of Korea; 8 Division of Pulmonology, Allergy and Critical Care Medicine Department of Internal Medicine Yongin Severance Hospital, Yonsei University College of Medicine Yongin Republic of Korea; 9 Division of Pulmonary and Critical Care Medicine Department of Internal Medicine Chungbuk National University Hospital, Chungbuk National University College of Medicine Cheong-Ju Republic of Korea; 10 Division of Tuberculosis Prevention and Control Korea Disease Control and Prevention Agency Osong Republic of Korea; 11 Zarathu Co Ltd Seoul Republic of Korea

**Keywords:** pulmonary tuberculosis, disease spectrum, timely diagnosis, patient delay, health care delay, risk factor, epidemiological disease, tuberculosis, treatment, TB, PTB disease spectrum, mortality, early diagnosis

## Abstract

**Background:**

Timely pulmonary tuberculosis (PTB) diagnosis is a global health priority for interrupting transmission and optimizing treatment outcomes. The traditional dichotomous time-divided approach for addressing time delays in diagnosis has limited clinical application because the time delay significantly varies depending on each community in question.

**Objective:**

We aimed to reevaluate the diagnosis time delay based on the PTB disease spectrum using a novel scoring system that was applied at the national level in the Republic of Korea.

**Methods:**

The Pulmonary Tuberculosis Spectrum Score (PTBSS) was developed based on previously published proposals related to the disease spectrum, and its validity was assessed by examining both all-cause and PTB-related mortality. In our analysis, we integrated the PTBSS into the Korea Tuberculosis Cohort Registry. We evaluated various time delays, including patient, health care, and overall delays, and their system-associated variables in line with each PTBSS. Furthermore, we reclassified the scores into distinct categories of mild (PTBSS=0-1), moderate (PBTBSS=2-3), and severe (PBTBSS=4-6) using a multivariate regression approach.

**Results:**

Among the 14,031 Korean patients with active PTB whose data were analyzed from 2018 to 2020, 37% (n=5191), 38% (n=5328), and 25% (n=3512) were classified as having a mild, moderate, and severe disease status, respectively, according to the PTBSS. This classification can therefore reflect the disease spectrum of PTB by considering the correlation of the score with mortality. The time delay patterns differed according to the PTBSS. In health care delays according to the PTBSS, greater PTB disease progression was associated with a shorter diagnosis period, since the condition is microbiologically easy to diagnose. However, with respect to patient delays, the change in elapsed time showed a U-shaped pattern as PTB progressed. This means that a remarkable patient delay in the real-world setting might occur at both apical ends of the spectrum (ie, in both mild and severe cases of PTB). Independent risk factors for a severe PTB pattern were age (adjusted odds ratio 1.014) and male sex (adjusted odds ratio 1.422), whereas no significant risk factor was found for mild PTB.

**Conclusions:**

Timely PTB diagnosis should be accomplished. This can be improved with use of the PTBSS, a simple and intuitive scoring system, which can be more helpful in clinical and public health applications compared to the traditional dichotomous time-only approach.

## Introduction

In 2020, an estimated 10 million cases of tuberculosis (TB) were reported worldwide [1]. In the Republic of Korea (ROK), the number of notified TB cases had long remained stable without a decrease [2,3]; however, the number of notified TB cases has decreased significantly in the last decade following continuous nationwide efforts [4,5]. Specifically, in 2021, the overall notification rate for incident TB cases (n=18,335) was 35.7/100,000 persons, which constituted a 53.6% decrease in the number of cases compared with those notified in 2011 (39,557 incident TB cases; notification rate 78.9/100,000 persons) [3]. As a low-TB-burden country, it is now an appropriate time to take the next step toward the goal of TB elimination in the ROK.

Timely TB diagnosis plays a crucial role in TB elimination by reducing the disease burden and preventing further community-based infections [6,7]. Numerous studies in various countries have focused on estimating the time delay for early TB diagnosis and treatment while identifying associated risk factors [7-19]. Previously, we also conducted research at the national level in the ROK, following a similar approach and drawing on references from previous studies [20]. However, we concluded that the binary time approach used in previous studies from other countries did not offer significant assistance in the context of the ROK. This is because the absolute value of the diagnostic delay time in the ROK was shorter than that observed in other countries. Therefore, we considered the clinical usefulness of this conventional dichotomous time-divided approach as limited for our context and attempted to approach the delayed diagnosis issue from a new perspective by focusing on the pulmonary TB (PTB) disease spectrum.

The “iceberg concept” has been applied to describe TB infection [21], illustrating that TB infection exists in varying quantities within the population. Furthermore, this concept helps to elucidate the disease spectrum, ranging from latent TB to clinical disease. According to the iceberg concept, two directions of effort are necessary to eliminate TB: the first involves moving from the bottom to the top of the iceberg in preventing progression from latent TB to active TB infection, whereas the second involves moving from the top to the bottom of the iceberg, reflecting the fact that early diagnosis and prompt treatment of active disease are crucial in preventing further community-based transmission of the infection.

In the ROK, active TB screening, including the progression from latent TB infection (LTBI) to clinical TB infection, especially in schools and military services, helped to significantly decrease the TB incidence among younger individuals in their 10s to 20s [4]. Active TB contact investigation and highly recommended treatment for LTBI are needed. Therefore, the remaining areas of early diagnosis of active TB at a national level are needed to assess and help establish TB control policies, while informing where efforts should be focused.

In this study, we investigated the diagnostic delay in timely PTB diagnosis; quantified the time-consuming processes contributing to patient, health care, and overall delays in access to TB treatment in the ROK based on the PTB disease spectrum; and compared demographic characteristics between cases diagnosed with mild versus severe disease. These data could reveal the epidemiological characteristics of PTB in the ROK, thereby informing the development of a real-world approach to PTB diagnosis that could provide important insights into the nature of the process wherein a patient develops symptoms and is finally diagnosed with PTB.

## Methods

### Korea TB Cohort and Recruitment

The Korea TB Cohort (KTBC) is a nationwide, prospective, and observational cohort comprising active TB cases from 172 public-private mix (PPM)–participating hospitals in 21 districts (>70% of all patients with TB in the ROK were treated in these PPM hospitals) since July 2018 [22]. Each patient with TB was notified, treated, and followed up every month until the completion of anti-TB treatment based on the national TB program. An investigation of the detailed data of the characteristics of active TB cases was planned to enable the establishment of a long-term plan in the future that shared the aim of an advanced National TB Elimination Project, which is operated by the Korean Academy of Tuberculosis and Respiratory Diseases under supervision of the Korea Disease Control and Prevention Agency (KDCA). The inclusion criteria for the KTBC include notified TB cases in all participating hospitals by the Korea National TB Surveillance System.

After enrollment of the KTBC, specialist TB nurses from each hospital conducted detailed interviews with patients with TB and completed standardized case-level forms. This process includes comprehensive investigations into patient information, including comorbidities, height, body weight, economic status, employment status, social status, education level, and symptoms. Additionally, data related to the program were gathered, including details about treatment initiation, discontinuation, termination, and adverse effects, along with mortality. The collected data were checked by regional and central data managers. Following a regional and central audit, a central statistical team analyzed and organized the data every quarter.

**Figure 1:**
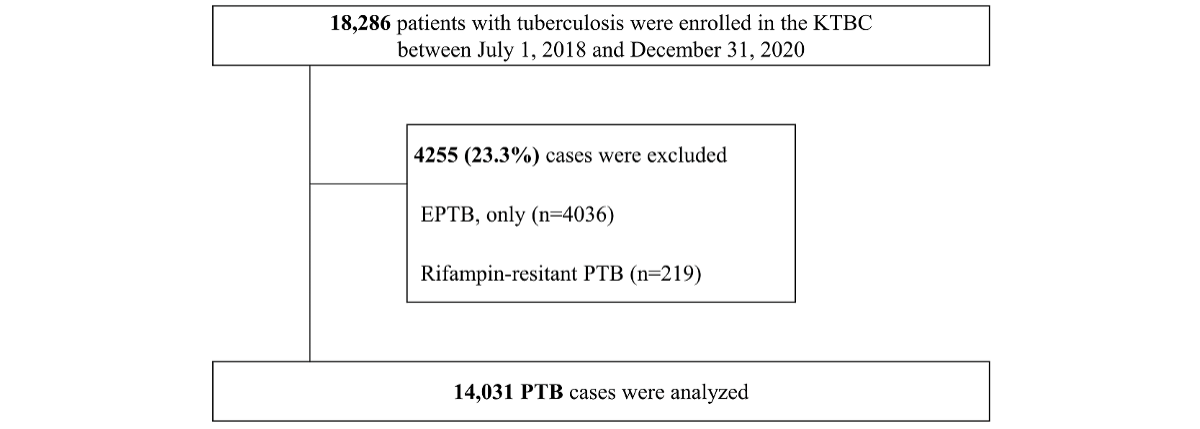
Flow diagram of the study design. EPTB: extrapulmonary tuberculosis; KTBC: Korea Tuberculosis Cohort; PTB: pulmonary tuberculosis.

Data collected from July 1, 2018, to December 31, 2020, were obtained from the KTBC. We included all PTB cases and excluded those with only extrapulmonary TB (EPTB) because the disease spectrum of EPTB could not be clearly determined. Furthermore, we excluded patients with rifampicin-resistant PTB to reduce heterogenicity when validating the developed Pulmonary Tuberculosis Spectrum Score (PTBSS) in comparing the disease spectrum of TB with outcomes (Figure 1).

### Definitions of Time Delays in the Diagnostic Pathway

We divided the PTB time interval into patient, health care, and overall delays [7]. A patient delay was defined as the duration between the onset of PTB-related symptoms and the first hospital visit. A health care delay was defined as the duration between the first hospital visit and initiation of anti-PTB treatment after a confirmed diagnosis of PTB. The overall delay was defined as the sum of the patient and health care delays.

### Determination of the PTBSS Based on the PTB Disease Spectrum

**Figure 2:**
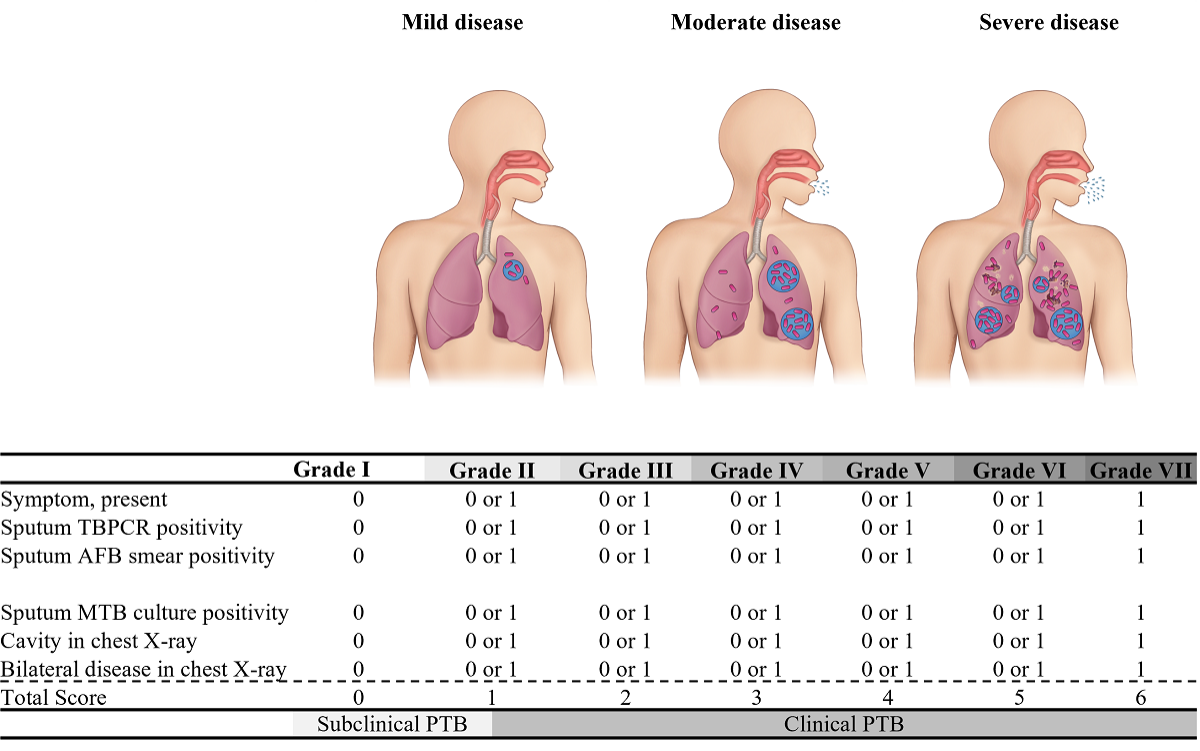
Determination of the Pulmonary Tuberculosis Spectrum Score (PTBSS). The PTBSS was calculated and distributed from 0 to 6 according to the presence of six clinical variables: symptoms, positive sputum in tuberculosis polymerase chain reaction (TBPCR), positive sputum in the acid-fast bacilli (AFB) smear test, sputum culture-positive Mycobacterium tuberculosis (MTB), cavitation on chest x-ray, and bilateral lung involvement of pulmonary tuberculosis (PTB) on chest x-ray scanning.

The PTBSS was designed based on previously published proposals for the PTB disease spectrum (Figure 2) [23-28]. Due to the lack of a true reference value that can accurately reflect the PTB disease spectrum, we identified factors that are commonly used in the diagnosis of PTB and can assess disease severity based on the epidemiological concept of PTB. We calculated the PTBSS according to six important variables: presence of symptoms, positive sputum in TB-polymerase chain reaction (PCR), positive sputum in an acid-fast bacilli (AFB) smear, sputum culture-positive *Mycobacterium tuberculosis*, cavitation detected on chest x-ray, and bilateral lung involvement of PTB on chest x-ray. However, since these six factors we identified and set were not weighted through statistical analysis, it may be challenging to view them as equally important. In addition, asymmetry can occur as the frequency of the manifestation of each factor can differ depending on the stage in the PTB disease spectrum. Therefore, for analysis, we proceeded by grouping in the following manner. Depending on the score assigned from 0 to 6 points, scores of 0-1, 2-3, and 4-6 were classified as mild, moderate, and severe disease, respectively.

### Validation of the PTBSS

While the PTBSS was designed based on a consensus around the disease spectrum, as supported by existing scientific evidence, it is necessary to validate the PTBSS within appropriate clinical and public health contexts. However, effective and specific biomarkers that are applicable in real-world settings have not been used, and these biomarkers cannot be implemented within the framework of the KTBC, which consists of clinical practice data from a real-world setting. Therefore, we sought to validate our results by comparing mortality rates according to the PTBSS. In accordance with the iceberg concept, disease progression could ultimately result in death. Hence, higher mortality could be associated with a longer disease duration. If the group with a high PTB score exhibits a high mortality rate, this group may have a longer disease period, which can be interpreted as the time elapsed from disease onset to diagnosis.

### Statistical Analysis

Categorical data are described as numbers and percentages, which were compared using the *χ*^2^ or Fisher exact test. Continuous variables are expressed as mean (SD) or median (IQR) for normal or skewed distributions, respectively, and were compared using the *t* test or Mann-Whitney *U* test. The Kaplan-Meier method was used to estimate the cumulative survival rates of active PTB according to the PTBSS during anti-TB treatment. To determine intergroup differences, survival curves were compared by means of log-rank tests, and hazard ratios with 95% CIs were estimated using Cox regression analysis. A multivariate logistic regression analysis by binary classification was used to identify independent risk factors for each mild and severe disease condition at the time of diagnosis, based on the PTBSS, as measured by the estimated odds ratio with 95% CI, including variables with *P*<.20 on univariate analysis [29]. All analyses were two-sided and statistical significance was set at *P*<.05. Statistical analyses were performed using R version 4.2.0 (R Foundation for Statistical Computing) and GraphPad Prism 9.4.

### Ethical Considerations

The Institutional Review Board of Hallym University Kangdong Sacred Heart Hospital approved the study protocol (approval number 2022-08-003-001) and waived the requirement for written informed consent from the participants because of the purely observational, noninterventional study design and analysis of anonymized patient data. The data are stored by the KDCA with authority to use as surveillance data for public health and research purposes.

## Results

### Participants

The flowchart in Figure 1 shows the recruitment of the study population after the exclusion of patients according to the above-described criteria. The distributions of these 14,031 patients with PTB according to the PTBSS are shown in Table 1.

The largest proportion of active cases of PTB were diagnosed as grades II to III (22.2% and 21.1%, respectively, according to a PTBSS of 1-2) and the smallest proportion of cases were diagnosed as grade VII (3.0% according to a PTBSS of 6). After the reclassification of severity based on the PTBSS, 37.0%, 38.0%, and 25.0% of patients with PTB were diagnosed with mild, moderate, and severe forms, respectively (Table 2). The detailed clinical characteristics of the enrolled 14,031 patients with PTB according to the PTBSS and reclassification of severity are presented in Table S1 in Multimedia Appendix 1.

**Table 1:** The number and proportions of patients with pulmonary tuberculosis (PTB) in the Korea Tuberculosis Cohort classified according to the PTB Spectrum Score (PTBSS).

Characteristics	Grade I	Grade II	Grade III	Grade IV	Grade V	Grade VI	Grade VII
PTBSS	0	1	2	3	4	5	6
Patients, n (%) (N=14,031)	2072 (14.8)	3119 (22.2)	2958 (21.1)	2370 (16.9)	1926 (13.7)	1167 (8.3)	419 (3.0)
Symptoms present, n (%)	0 (0.0)	1637 (52.5)	2136 (72.2)	1919 (81.0)	1744 (90.6)	1129 (96.7)	419 (100.0)
Sputum TB PCR^a^ positivity, n (%)	0 (0.0)	173 (5.5)	756 (25.6)	1445 (61.0)	1732 (89.9)	1114 (95.5)	419 (100.0)
Sputum AFB^b^ smear-positive, n (%)	0 (0.0)	0 (0.0)	201 (6.8)	555 (23.4)	1301 (67.5)	1094 (93.7)	419 (100.0)
Sputum MTB^c^ culture-positive, n (%)	0 (0.0)	743 (23.8)	1608 (54.4)	1818 (76.7)	1793 (93.1)	1137 (97.4)	419 (100.0)
Cavity in chest x-ray, n (%)	0 (0.0)	180 (5.8)	345 (11.7)	414 (17.5)	374 (19.4)	499 (42.8)	419 (100.0)
Bilateral disease in chest x-ray, n (%)	0 (0.0)	386 (12.4)	870 (29.4)	959 (40.5)	760 (39.5)	862 (73.9)	419 (100.0)

^a^TB PCR: tuberculosis polymerase chain reaction.

^b^AFB: acid-fast bacilli.

^c^MTB: *Mycobacterium tuberculosis*.

**Table 2:** Patients with pulmonary tuberculosis (PTB) classified according to disease severity (N=14,031).

Characteristics	Mild	Moderate	Severe
PTB Spectrum Score	0 to 1	2 to 3	4 to 6
Patients, n (%)	5191 (37.0)	5328 (38.0)	3512 (25.0)
Symptom present, n (%)	1637 (31.5)	4055 (76.1)	3292 (93.7)
Sputum TB PCR^a^ positivity, n (%)	173 (3.3)	2202 (41.3)	3265 (93.0)
Sputum AFB^b^ smear-positive, n (%)	0 (0.0)	756 (14.2)	2814 (80.1)
Sputum MTB^c^ culture-positive, n (%)	743 (14.3)	3426 (64.3)	3349 (95.4)
Cavity in chest x-ray, n (%)	180 (3.5)	759 (14.2)	1292 (36.8)
Bilateral disease in chest x-ray, n (%)	386 (7.4)	1829 (34.3)	2041 (58.1)

^a^TB PCR: tuberculosis polymerase chain reaction.

^b^AFB: acid-fast bacilli.

^c^MTB: *Mycobacterium tuberculosis*.

### Validation of the PTBSS Based on Survival Analysis

**Figure 3:**
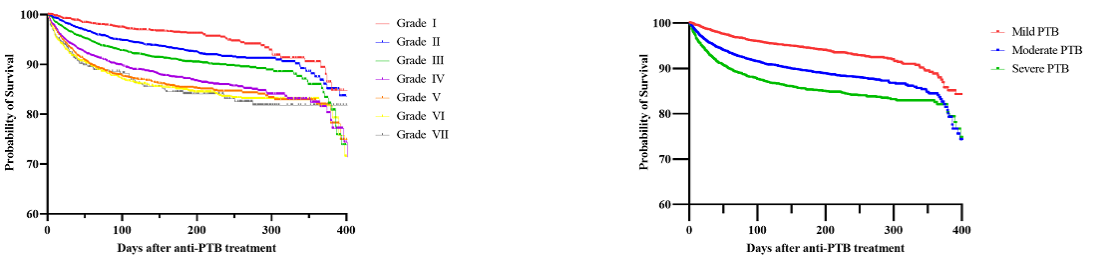
Kaplan-Meier survival probability curves of all-cause mortality for patients with active pulmonary tuberculosis (PTB) according to the Pulmonary Tuberculosis Spectrum Score (PTBSS; left curve, seven groups) and disease severity classified based on the PTBSS (right curve, three groups).

We evaluated the cumulative survival rate of KTBC patients with respect to both all-cause and PTB-related mortality, according to both the PTBSS itself and reclassified disease severity based on the PTBSS. With regard to all-cause mortality (Figure 3, Table S2 in Multimedia Appendix 1), survival rates were higher in patients with a low PTBSS and mild disease than in those with high scores and moderate-to-severe disease (Kaplan-Meier analysis, log-rank test *P*<.001). The overall cumulative mortality rates during anti-TB treatment for active PTB were 4.9% (grade I, score of 0), 8.0% (grade II, score of 1), 11.0% (grade III, score of 2), 14.3% (grade IV, score of 3), 14.6% (grade V, score of 4), 15.6% (grade VI, score of 5), and 14.6% (grade VII, score of 6). The overall cumulative mortality rates during anti-TB treatment for active PTB were 6.8% (mild disease), 12.4% (moderate disease), and 14.9% (severe disease).

**Figure 4:**
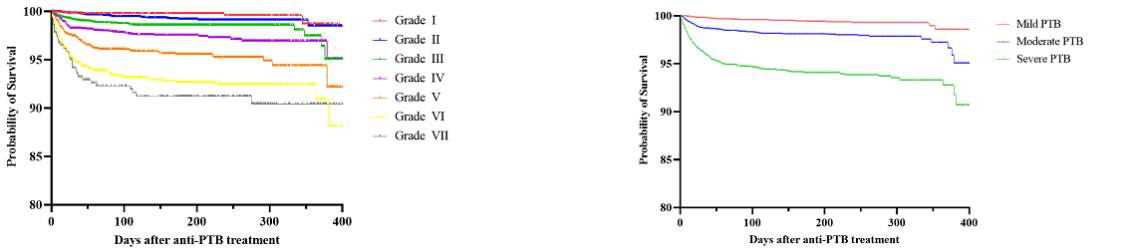
Kaplan-Meier survival probability curves of pulmonary tuberculosis (PTB)-related mortality according to the Pulmonary Tuberculosis Spectrum Score (PTBSS; left curve, seven groups) and disease severity classified based on the PTBSS (right curve, three groups).

With respect to PTB-related mortality (Figure 4, Table S3 in Multimedia Appendix 1), survival rates were higher in patients with a low PTBSS and mild disease compared with those in the high score and moderate-to-severe disease groups (*P*<.001). The PTB-related cumulative mortality rates during anti-TB treatment for active PTB were 0.4% (grade I), 0.8% (grade I), 1.5% (grade III), 2.7% (grade IV), 4.2% (grade V), 7.3% (grade VI), and 8.1% (grade VII). The overall cumulative mortality rates during anti-TB treatment for active PTB were 0.7% (mild disease), 2.0% (moderate disease), and 5.7% (severe disease). Considering the correlation between the PTBSS and mortality, the PTBSS can be considered to reflect the natural course of PTB.

### Time Delays According to the PTBSS in the Diagnostic Pathway

**Figure 5:**
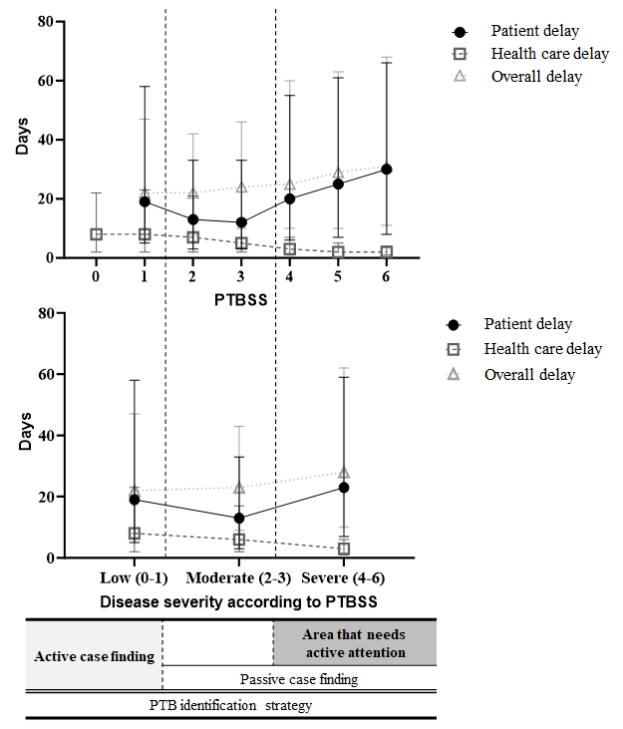
Patient, health care, and overall delays according to the PTBSS (top) and disease severity classified according to the PTBSS. Patient, health care, and overall time delays are presented as the median with IQR. PTB: pulmonary tuberculosis; PTBSS: Pulmonary Tuberculosis Spectrum Score.

After evaluating whether the PTBSS reflects the natural course of PTB, we checked all possible types of time delays according to the scores (Figure 5). As the PTBSS increased, the health care delay gradually decreased. However, the patient delay decreased with an increase in the PTBSS from 1 to 3 and then increased again from a score of 4 and above. The overall delay, as the sum of the health care and patient delays, gradually increased as the PTBSS increased. When including time delays in the diagnostic pathway and classifying them on a spectrum from mild to severe disease, health care and overall delays increased with increased disease severity. However, the patient delay exhibited a U-shaped pattern (Figure 5). This means that different patient-related time delays in the real-world setting, ranging from symptom onset of PTB to the first hospital visit, appeared at both extremes (ie, in the mild and severe forms) of PTB. This further means that two different approaches are required to reduce the patient delay.

Furthermore, we conducted comparative analyses to verify the impact of each factor included in the construction of the PTBSS on patient delay, health care delay, and overall delay. The results are presented in Table 3. Patients without symptoms exhibited differences in health care delay, likely indicating that symptomatic patients receive expedited testing in hospitals. Both sputum TB PCR positivity and AFB smear positivity were associated with differences in both patient and health care delays. In contrast, a positive *Mycobacterium tuberculosis* (MTB) culture in sputum did not exhibit any significant difference in either the patient or health care delay. The presence of cavitation on chest x-rays appeared to affect both patient and health care delays similarly. However, while bilateral lung involvement seemed to reduce the health care delay, it did not show a significant impact on the patient delay.

**Table 3:** Patient, health care, and overall delays according to each scoring factor of the Pulmonary Tuberculosis Spectrum Score.

Scoring factor		Patients, n (%)	Patient delay, median (IQR)	Health care delay, median (IQR)	Overall delay, median (IQR)
**Symptoms**					
	Without symptoms	5047 (36.0)	N/A^a^	8.0 (2.0-22.0)	8.0 (2.0-22.0)
	With symptoms	8984 (64.0)	16.0 (5.0-46.0)	4.0 (1.0-11.0)	28.0 (10.0-58.0)
	*P* value	N/A	N/A	<.001	<.001
**Sputum TB PCR^b^**					
	Negative	8392 (59.8)	15.0 (4.0-41.5)	8.0 (2.0-23.0)	21.0 (7.0-42.0)
	Positive	5639 (40.2)	19.0 (5.0-50.0)	3.0 (1.0-7.0)	21.0 (8.0-44.0)
	*P* value	N/A	<.001	<.001	.007
**Sputum AFB^c^ smear**					
	Negative	3570 (25.4)	14.0 (4.0-37.0)	7.0 (2.0-20.0)	19.0 (7.0-40.0)
	Positive	10,461 (74.6)	24.0 (6.0-60.0)	3.0 (1.0-6.0)	28.0 (9.0-62.0)
	*P* value	N/A	<.001	<.001	<.001
**Sputum MTB^d^ culture**					
	Negative	5307 (37.8)	16.0 (4.0-56.0)	6.0 (1.0-14.0)	14.0 (6.0-36.0)
	Positive	8724 (62.2)	16.0 (5.0-39.0)	5.0 (2.0-14.0)	24.0 (9.0-48.0)
	*P* value	N/A	.62	.59	<.001
**Cavity in chest x-ray**					
	Negative	11,800 (84.1)	15.0 (4.0-39.0)	6.0 (2.0-16.0)	19.0 (7.0-40.0)
	Positive	2231 (15.9)	26.0 (7.0-61.0)	3.0 (1.0-8.0)	30.0 (11.0-62.0)
	*P* value	N/A	<.001	<.001	<.001
**Extent of PTB^e^**					
	Unilateral	9775 (69.7)	16.0 (5.0-41.0)	6.0 (2.0-16.0)	19.0 (7.0-40.0)
	Bilateral	4256 (30.3)	17.0 (4.0-55.0)	4.0 (1.0-10.0)	27.0 (9.8-59.0)
	*P* value	N/A	.12	<.001	<.001

^a^N/A: not applicable.

^b^TB PCR: tuberculosis polymerase chain reaction.

^c^AFB: acid-fast bacilli.

^d^MTB: *Mycobacterium tuberculosis*.

^e^PTB: pulmonary tuberculosis.

### Risk Factors for a Diagnosis of Mild or Severe Disease Based on the PTBSS

The independent risk factors associated with mild or severe disease at the time of diagnosis were investigated (Table 4). In the multivariate analysis adjusted for potential confounding factors, age and male sex were independently associated with severe PTB in patient delay. The significant difference in symptoms such as cough and/or sputum, dyspnea, and hemoptysis may be attributed to the progression of PTB itself and may be unrelated to the late patient delay of severe PTB. However, no factor was significantly associated with patient delay for mild PTB.

**Table 4:** Univariable and multivariable logistic regression models for the probability of diagnosing severe or mild disease according to the Pulmonary Tuberculosis Spectrum Score.

Variable			Univariable logistic regression			Multivariable logistic regression	
		Odds ratio (95% CI)		*P* value	Adjusted odds ratio (95% CI)		*P* value
**Severe PTB^a^**							
	Age	1.006 (1.004-1.008)		<.001	1.014 (1.002-1.027)		.03
	Age≥65 years	1.067 (0.988-1.151)		.10	0.699 (0.430-1.137)		.15
	Sex (male vs female)	1.313 (1.211-1.424)		<.001	1.422 (1.027-1.969)		.03
	Poor economic status	1.364 (1.111-1.675)		.003	1.550 (0.992-2.419)		.05
	BMI	0.903 (0.893-0.914)		<.001	0.920 (0.882-0.959)		<.001
	Comorbidity of chronic kidney disease	0.796 (0.630-1.005)		.05	0.308 (0.116-0.817)		.02
	Symptom of cough and/or sputum	4.167 (3.842-4.519)		<.001	7.197 (1.999-25.908)		.003
	Symptom of dyspnea	2.050 (1.862-2.258)		<.001	3.804 (1.046-13.838)		.04
	Symptom of hemoptysis	1.725 (1.469-2.027)		<.001	5.121 (1.361-19.275)		.02
	Symptom of fever	1.962 (1.766-2.181)		<.001	3.802 (1.044-13.840)		.04
	Symptom of malaise	2.973 (2.573-3.435)		<.001	7.010 (1.801-27.292)		.005
	Symptom of weight loss	4.009 (3.545-4.532)		<.001	6.076 (1.584-23.307)		.009
	History of TB^b^ infection	0.951 (0.856-1.057)		.35	0.576 (0.391-0.848)		.005
	With EPTB^c^	0.738 (0.640-0.851)		<.001	0.577 (0.345-0.964)		.04
**Mild PTB**							
	Age	0.985 (0.983-0.987)		<.001	0.982 (0.971-0.994)		.002
	Age≥65 years	0.654 (0.611-0.701)		<.001	1.138 (0.731-1.770)		.57
	BMI	1.076 (1.065-1.087)		<.001	1.068 (1.028-1.110)		<.001
	Comorbidity of chronic heart disease	0.609 (0.518-0.717)		<.001	0.478 (0.253-0.903)		.02

^a^PTB: pulmonary tuberculosis.

^b^TB: tuberculosis.

^c^EPTB: extrapulmonary tuberculosis.

## Discussion

### Principal Results

The time elapsed from the onset of PTB to diagnosis could be heterogeneous depending on personal, cultural, and health system situations. For effective TB control, the best solution needs to be identified according to each situation. We have previously recognized that a time-only approach is insufficient for the timely diagnosis of active PTB. In this study, we developed a new approach based on the disease spectrum of PTB, deviating from the perspective that has been studied based on the time delay itself.

The main findings of this study are as follows. First, the PTBSS could reflect the disease spectrum of PTB by considering the correlation of the score with mortality. Second, the pattern of time delays differed according to the PTBSS. In health care delays according to the PTBSS, greater PTB progression was associated with a shorter diagnosis period, because the condition is microbiologically easy to diagnose. However, with respect to patient delays, the change in elapsed time showed a U-shaped pattern as PTB progressed. This means that a remarkable patient delay in the real-world setting might occur at both apical ends of the spectrum (ie, in mild and severe PTB). This further means that two different approaches are required to reduce the patient delay. Third, the independent risk factors of a late visit to a medical institution as patient delay factors were age and male sex in the severe form of PTB. In contrast, there were no significant risk factors for mild PTB. Considering the natural course of PTB infection within the population, our approach could be helpful for a diagnostic strategy of active PTB involving passive case finding for the severe form and active case finding for mild forms [26] (Figure 5).

### Comparison With Prior Work

A diagnostic approach for the timely diagnosis and subsequent treatment of PTB is essential to reduce ongoing transmission in the community and PTB-related morbidity and mortality [30,31]. This approach is composed of two different types of time delays: patient and health care delays [7]. Both patient and health care delays have different medical and public health implications. Patient delay is the major determining time period for the total duration of the PTB diagnostic pathway, and is associated with infectiousness due to long-term exposure to others in the community and poor outcomes due to disease progression [11,20]. In contrast, the health care delay is the period immediately preceding the diagnosis of PTB, which is characterized by the highest infectious state in the disease course of PTB and is associated with in-hospital transmission to health care workers or other patients [32]. To date, studies have investigated timely diagnosis and related risk factors according to the time dichotomy [8,9,11,12,14,20,33]. However, this approach has inherent limitations for general application because the time delay and risk factors can vary greatly depending on the environments within each country [7,9,10,12,13,15,19,33]. Among the types of time delays, the patient delay is greatly influenced by the culture of each country, such as the perception of disease, personal circumstances, and medical policy. Health care delay will be affected by the specialty of the health care provider and the medical resources of each country. Therefore, it was difficult to identify consistent risk factors in previous studies.

PTB can be presented as a dynamic spectrum along with pathophysiology resulting from bacterial progression and associated changes in the host response, which is distinct from the binary simple classification of active and latent TB infection [25,27]. To reach the goal of TB elimination, a promising approach that possesses the above-mentioned characteristics is essential for a timely diagnosis of PTB and includes recognizing the development of subclinical PTB from an LTBI state, early detection of active PTB from subclinical PTB, and timely differential diagnosis of severe PTB from mild PTB [24,27,28,34]. The insights and perspectives on PTB as a spectrum in a disease state are becoming increasingly accepted, leading to new diagnostic approaches for different stages of the disease spectrum. However, in clinical practice, in a real-world setting, this is considerably more complicated than what is usually expected because of the limited evidence on predictive biomarkers for disease progression within active PTB [24]. Therefore, a simple and accessible approach is needed from both clinical and public health perspectives. Thus, we attempted to design the PTBSS for clinical and public use, which is expected to be easily applied in clinical and public health practice, as the PTBSS consists of variables that are widely used in real-world settings.

We aimed to validate this proposed and devised scoring system by examining the correlation of the PTBSS and the mortality rate during the treatment of PTB. The results revealed that for all-cause mortality, there was no significant difference in the Kaplan-Meier curve and hazard ratio between high-grade scores (eg, grade IV to VII), while clear distinctions were observed when grouping into mild, moderate, and severe disease categories. Conversely, for PTB-related mortality, significant differences were observed either when considering each PTBSS grade or when classifying patients into three distinct groups. We believe that the PTBSS, as an operational definition to predict the disease spectrum of PTB, reflects PTB-related mortality relatively better than all-cause mortality, which indirectly might demonstrate its excellence in reflecting the disease spectrum of PTB. Among 1538 deaths (10.9%) recorded after a confirmed diagnosis and treatment initiation in the KTBC, 342 (2.4%) were related to PTB, while 1196 (8.5%) were due to other causes. Most PTB-related deaths were a result of respiratory failure (n=324, 94.7%), with secondary ischemic heart disease accounting for 20 deaths (5.4%) and death from massive hemoptysis for 5 cases (1.5%).

As shown in Table 3, we conducted comparative analyses to assess the impact of various factors on each delay in PTBSS construction. Asymptomatic patients experienced health care delays, suggesting that hospitals may expedite testing for those with symptoms. A positive sputum TB PCR result was linked to both patient and health care delays; health care providers might quickly decide on treatment for TB PCR–positive patients, thereby reducing health care delays. However, TB PCR positivity may also lead to longer patient delays due to later hospital visits after symptom onset. AFB smear positivity of sputum also affected both delays, likely for similar reasons. Conversely, positive MTB cultures in sputum did not influence either delay, possibly because treatment typically starts when PTB is suspected clinically rather than waiting for culture results. Cavitation on chest x-rays was associated with delays in a similar way to TB PCR and AFB smear positivity. Bilateral lung involvement seemed to shorten the health care delay but not patient delay, as health care providers might act quickly in such cases, while the patient’s perception of the disease onset—whether unilateral or bilateral—might not affect their decision to seek care. With this novel approach, we determined at what level of severity, the degree of patient delay, and the degree of health care delay that cases of PTB are diagnosed in the ROK based on the severity of PTB. This new perspective will help to change the classic perspective of active PTB and establish a more specific and realistic PTB elimination policy. We confirmed that the patient delay of PTB in the ROK was not linearly correlated, such as an increase in time with PTB progression.

The definition of patient delay, which refers to the time taken by a patient to visit a health care facility after the onset of symptoms, can lead to discrepancies in the measured period and the associated risk factors due to the inherent inaccuracy of a patient’s subjective symptom onset. Because of these limitations, numerous studies have shown that while the health care delay remains somewhat consistent, the patient delay significantly varies depending on each country in question. Factors such as each society’s medical infrastructure, economic conditions, perspectives on the disease, and language barriers are complexly intertwined, suggesting that an integrated approach that a society can employ is necessary. From this standpoint, we believe that using objective factors, rather than just relying on a patient’s subjective symptom onset, and employing the PTBSS, which can reflect the natural progression of PTB, could be helpful in understanding the factors causing TB diagnostic delays in each society.

According to our study, older individuals with PTB were more likely to be diagnosed with advanced disease, possibly because they were unaware of TB-related symptoms because of their preexisting comorbidities or because they considered the possibility of diseases other than PTB. Thus, older adults were probably more likely to visit medical facilities late after symptom onset. Moreover, even if older people visit a medical institution, the diagnosis of PTB can be delayed because of the possibility of other diseases. Furthermore, male patients are likely to be diagnosed with advanced disease. This is consistent or inconsistent with the results of previous observational studies conducted in different countries [8,11,12,14]. Thus, more research is needed.

### Limitations

This study has some limitations. First, this study was performed in the ROK, a country with a high level of medical resources, the highest rate of health care use among the Organisation for Economic Co-operation and Development (OECD) countries, an aging population, and a low prevalence of HIV. Therefore, our analysis may have overestimated or underestimated the prevalence of PTB. Second, the patient delay could be affected by recall bias because this factor is determined by the patient’s own symptom description. Third, we only evaluated the disease spectrum using clinical variables, without specific immunological or bacteriological data, due to a lack of laboratory-based information. Fourth, the timing data for patient delay may vary between passive and active case-finding. Active case-finding might result in a shorter patient delay compared to passive case-finding. Nevertheless, we believe that a patient delay would primarily occur in symptomatic patients in the ROK, where a nationwide health insurance system enables citizens to access medical services despite economic constraints. Koreans are more likely to seek medical care at facilities compared to citizens from other OECD countries due to the ease of accessibility to medical services. However, we acknowledge that despite these favorable circumstances, there could be instances where some symptomatic patients might not seek medical attention due to personal reasons. To gain a deeper understanding of these diagnostic situations, the KTBC made the decision, in March 2022 during a meeting with the KDCA, to investigate the reasons for hospital visits. As a result, we plan to consider these factors and incorporate relevant analyses in our future follow-up studies.

### Conclusions

A diagnostic approach for the timely diagnosis of PTB would be improved if based on the disease spectrum rather than the traditional dichotomous time-only approach. The PTBSS, as a simple and intuitive scoring system, could facilitate clinical and public approaches for TB detection and elimination specific to the context of each country.

## Appendix

Multimedia Appendix 1 Characteristics and time delays of participants according to Pulmonary Tuberculosis Spectrum Score (Table S1); Cox proportional hazard model for all-cause mortality (Table S2); Cox proportional hazard model for pulmonary tuberculosis–related mortality (Table S3).

## Abbreviations

AFB: acid-fast bacilli
EPTB: extrapulmonary tuberculosis
KDCA: Korea Disease Control and Prevention Agency
KTBC: Korean Tuberculosis Cohort
LTBI: latent tuberculosis infection
MTB: Mycobacterium tuberculosis
OECD: Organisation for Economic Co-operation and Development
PCR: polymerase chain reaction
PPM: public-private mix
PTB: pulmonary tuberculosis
PTBSS: Pulmonary Tuberculosis Spectrum Score
ROK: Republic of Korea
TB: tuberculosis

## Data Availability

The data that support the findings of this study are available upon request from the Korea Disease Control and Prevention Agency (KDCA). These data are not publicly available because of privacy or ethical restrictions. The Korea Tuberculosis Cohort data are currently being organized and there is a plan to make these data available to interested researchers in 2024, provided that prior permission is obtained from the KDCA.
