# Supplementary material for: Timely Pulmonary Tuberculosis Diagnosis Based on the Epidemiological Disease Spectrum: Population-Based Prospective Cohort Study in the Republic of Korea
Source: JMIR Public Health Surveill. 2024 Apr 1;10:e47422. doi: 10.2196/47422 (PMC11019417; doi:10.2196/47422)
Supplement: Multimedia Appendix 1 [file publichealth_v10i1e47422_app1.docx]

**Table S1**. General characteristics and time delays of 14,031 participants in the Korea Tuberculosis Cohort according to the Pulmonary Tuberculosis Spectrum Score–based classification of disease severity.

| Variables | | Mild PTB^a^ (score=0-1) (n=5191) | Moderate PTB (score=2-3) (n=5328) | Severe PTB (score=4-6) (n=3512) | *P* value |
| --- | --- | --- | --- | --- | --- |
| Age (years), mean (SD) | | 58.6 (18.8) | 64.2 (18.7) | 63.6 (17.9) | <.001 |
| Age≥65 years, n (%) | | 2163 (41.7) | 2893 (54.3) | 1734 (49.4) | <.001 |
| Male sex, n (%) | | 3130 (60.3) | 3364 (63.1) | 2386 (67.9) | <.001 |
| BMI (kg/m^2^), mean (SD) | | 21.8 (3.3) | 21.3 (3.4) | 20.5 (3.4) | <.001 |
| **Body weight classification** | | | | | <.001 |
|  | Severely underweight (BMI<16), n (%) | 139 (2.7) | 241 (4.5) | 292 (8.4) |  |
|  | Underweight (BMI 16-18.4), n (%) | 599 (11.6) | 797 (15.0) | 683 (19.5) |  |
|  | Normal weight (BMI 18.5-24.9), n (%) | 3678 (71.1) | 3589 (67.6) | 2211 (63.3) |  |
|  | Overweight (BMI 25-29.9), n (%) | 682 (13.2) | 613 (11.6) | 285 (8.2) |  |
|  | Obese (BMI ≥30), n (%) | 75 (1.4) | 66 (1.2) | 23 (0.7) |  |
| Poor economic status, n (%) | | 135 (2.6) | 169 (3.2) | 137 (3.9) | .003 |
| Rural area of residence, n (%) | | 1220 (23.5) | 1386 (26.0) | 864 (24.6) | .01 |
| Living alone, n (%) | | 1986 (38.3) | 2162 (40.6) | 1497 (42.6) | <.001 |
| Educational status below the compulsory education level, n (%) | | 211 (34.0) | 274 (42.1) | 174 (42.2) | .004 |
| Foreign national, n (%) | | 226 (4.4) | 183 (3.4) | 114 (3.2) | .01 |
| Former or current smoker, n (%) | | 2140 (41.3) | 2194 (41.2) | 1684 (48.0) | <.001 |
| Heavy alcohol intake, n (%) | | 1839 (39.7) | 1773 (36.3) | 1340 (41.3) | <.001 |
| **Symptoms** | | | | | |
|  | Symptom score (0-70), mean (SD) | 0.41 (0.7) | 1.1 (0.9) | 1.5 (0.9) | <.001 |
|  | Cough and/or sputum, n (%) | 945 (18.2) | 2442 (45.8) | 2333 (66.4) | <.001 |
|  | Dyspnea, n (%) | 334 (6.4) | 1038 (19.5) | 826 (23.5) | <.001 |
|  | Chest pain, n (%) | 205 (3.9) | 407 (7.6) | 224 (6.4) | <.001 |
|  | Hemoptysis, n (%) | 139 (2.7) | 301 (5.6) | 246 (7.0) | <.001 |
|  | Fever, n (%) | 278 (5.4) | 815 (15.3) | 651 (18.5) | <.001 |
|  | Malaise, n (%) | 96 (1.8) | 320 (6.0) | 383 (10.9) | <.001 |
|  | Weight loss, n (%) | 159 (3.1) | 376 (7.1) | 621 (17.7) | <.001 |
| **Comorbidities, n (%)** | | | | | |
|  | Any comorbidity | 2949 (56.8) | 3440 (64.6) | 2155 (61.4) | <.001 |
|  | Diabetes mellitus | 980 (18.9) | 1149 (21.6) | 885 (25.2) | <.001 |
|  | Chronic pulmonary disease | 217 (4.2) | 392 (7.4) | 177 (5.0) | <.001 |
|  | Chronic heart disease | 210 (4.0) | 380 (7.1) | 192 (5.5) | <.001 |
|  | Chronic liver disease | 127 (2.4) | 134 (2.5) | 87 (2.5) | .98 |
|  | Chronic kidney disease | 148 (2.9) | 196 (3.7) | 92 (2.6) | .008 |
|  | Neuropsychiatric disease | 391 (7.5) | 610 (11.4) | 337 (9.6) | <.001 |
|  | Malignancy | 628 (12.1) | 489 (9.2) | 229 (6.5) | <.001 |
|  | Autoimmune disease | 45 (0.9) | 63 (1.2) | 41 (1.2) | .22 |
| **Medications, n (%)** | | | | | |
|  | Long-term steroid use | 17 (0.3) | 22 (0.4) | 19 (0.5) | .31 |
|  | TNF^b^-α blocker | 6 (0.1) | 7 (0.1) | 3 (0.1) | .90 |
| Gastrectomy or jejunostomy | | 54 (1.0) | 63 (1.2) | 36 (1.0) | .71 |
| Organ transplantation | | 13 (0.3) | 19 (0.4) | 13 (0.4) | .53 |
| HIV infection | | 15 (0.3) | 17 (0.3) | 7 (0.2) | .57 |
| History of tuberculosis infection | | 784 (15.1) | 906 (17.0) | 541 (15.4) | .02 |
| PTB with EPTB^c^ | | 387 (7.5) | 626 (11.7) | 256 (7.3) | <.001 |
| **Delays** | | | | | |
|  | Patient delay, mean (SD) | 51.3 (102.8) | 36.9 (80.6) | 47.6 (83.6) | <.001 |
|  | Patient delay, median (IQR) | 19.0 (5.0-58.0) | 13.0 (3.0-33.0) | 23.0 (7.0-59.0) |  |
|  | Health care delay, mean (SD) | 19.3 (37.1) | 14.3 (30.2) | 6.9 (25.1) | <.001 |
|  | Health care delay, median (IQR) | 8.0 (2.0-23.0) | 6.0 (2.0-17.0) | 3.0 (1.0-6.0) |  |
|  |  |  |  |  |  |
|  | Overall delay, mean (SD) | 33.5 (69.3) | 42.3 (75.8) | 50.9 (84.1) | <.001 |
|  | Overall delay, median (IQR) | 14.0 (5.0-36.0) | 23.0 (9.0-43.0) | 28.0 (10.0-62.0) |  |
| Hospitalization during antituberculosis therapy, n (%) | | 2184 (42.1) | 3076 (57.7) | 2332 (66.4) | <.001 |
| Number of hospital visits, mean (SD) | | 7.8 (5.0) | 7.7 (5.2) | 7.9 (5.4) | .60 |

^a^PTB: pulmonary tuberculosis.

^b^TNF: tumor necrosis factor.

^c^EPTB: extrapulmonary tuberculosis.

**Table S2.** Cox proportional hazard model for all-cause mortality during antituberculosis treatment according to the Pulmonary Tuberculosis Spectrum Score (PTBSS) and disease severity.

| Disease severity | | Hazard ratio (95% CI) | *P* value |
| --- | --- | --- | --- |
| **Mild (PTBSS=0-2)** | | Reference | —^a^ |
|  | 0 | Reference | — |
|  | 1 | 1.635 (1.284-2.083) | <.001 |
|  | 2 | 2.139 (1.688-2.710) | <.001 |
| **Moderate (PTBSS=3-4)** | | 1.829 (1.593-2.101) | <.001 |
|  | 3 | 3.000 (2.376-3.790) | <.001 |
|  | 4 | 3.342 (2.637-4.236) | <.001 |
| **Severe (PTBSS=5-6)** | | 2.482 (2.155-2.860) | <.001 |
|  | 5 | 3.545 (2.753-4.565) |  |
|  | 6 | 3.430 (2.475-4.754) | <.001 |

^a^Not applicable.

**Table S3.** Cox proportional hazard model for pulmonary tuberculosis–related mortality during antituberculosis treatment according to the Pulmonary Tuberculosis Spectrum Score (PTBSS) and disease severity.

| Disease severity | | Hazard ratio (95% CI) | *P* value |
| --- | --- | --- | --- |
| **Mild (PTBSS=0-2)** | | Reference | —^a^ |
|  | 0 | Reference | — |
|  | 1 | 2.328 (1.003-5.403) | .06 |
|  | 2 | 4.211 (1.883-9.414) | <.001 |
| **Moderate (PTBSS=3-4)** | | 3.156 (2.103-4.736) | <.001 |
|  | 3 | 7.460 (3.397-16.380) | <.001 |
|  | 4 | 12.929 (5.963-28.031) | <.001 |
| **Severe (PTBSS=5-6)** | | 9.642 (6.593-14.101) | <.001 |
|  | 5 | 21.467 (9.905-46.524) | <.001 |
|  | 6 | 25.456 (11.258-57.561) | <.001 |

^a^Not applicable.
